# PROPSY: a new strategy to implement precision psychiatry in France

**DOI:** 10.1192/j.eurpsy.2023.31

**Published:** 2023-07-19

**Authors:** P. M. Llorca

**Affiliations:** CHU, Clermont-Ferrand, France

## Abstract

**Abstract:**

Over a 5-year program-project, focusing on 4 of the most disabling disorders (i.e. Bipolar Disorder, Major Depressive Disorders, Schizophrenia, and Autism Spectrum Disorders), PROPSY’s ambition is to bring solutions for precision medicine in psychiatry. This ambition requires overcoming multiple challenges: (i) to discover prognostic and stratification biomarkers, (ii) to better understand underlying causes and mechanisms, (iii) to develop targeted therapeutic strategies (iii) to reduce stigma and false representation, (iv) to reduce the direct and indirect economic costs. To tackle these challenges, PROPSY is an inclusive program project composed of 5 operational work packages. PROPSY will also implement this knowledge into clinical practice, in a truly learning healthcare system, and increase awareness with Patients’ Associations to reach out to civil society. This research effort in precision psychiatry aims to strengthen the coordination of the French workforce along with international collaborations in connection with users and policymakers

**Disclosure of Interest:**

None Declared

